# miR-199a-5p suppresses human bladder cancer cell metastasis by targeting CCR7

**DOI:** 10.1186/s12894-016-0181-3

**Published:** 2016-11-04

**Authors:** Mi Zhou, Shuai Wang, Linyi Hu, Feng Liu, Qi Zhang, Dahong Zhang

**Affiliations:** Department of Urology, Zhejiang Provincial People’s Hospital, 158 Shangtang Road, Hangzhou, Zhejiang Province 310014 People’s Republic of China

**Keywords:** Bladder cancer, Metastasis, CCR7, miR-199a-5p

## Abstract

**Background:**

C-C chemokine receptor type 7 (CCR7) overexpression correlated with lymphatic metastasis and poor prognosis is a major obstacle to bladder cancer treatment. Recent studies have revealed that miR-199a-5p was significantly abnormal expressed in several solid tumors and functioned as oncogene or tumor suppressor. This study was aimed to further investigate the effects of miR-199a-5p on the cell metastasis mediated by CCR7 in bladder cancer.

**Methods:**

Quantitative Real Time PCR (qRT-PCR) was firstly performed to identified the expression of miR-199a-5p and CCR7 in human bladder cancer samples and cell lines. Following that, the effects of miR-199a-5p on cell migratory and invasive activities were assessed by wound healing and Matrigel invasion assays, respectively. Finally, luciferase reporter assay and western blot were employed to investigate whether CCR7 could directly interact with miR-199a-5p.

**Results:**

miR-199a-5p downregulation and CCR7 upregulation were firstly observed in bladder cancer samples and cell lines. In addition, both miR-199a-5p downregulation and CCR7 upregulation were significantly involved in bladder cancer clinicopathological features. Moreover, overexpression of miR-199a-5p could inhibit baldder cancer cell migration and invasion. miR-199a-5p was confirmed to be able to target the 3′ untranslated region (UTR) of CCR7 and regulate the expression of CCR7, Matrix metalloproteinases 9 (MMP-9) and Epithelial-Mesenchymal Transition (EMT)-related proteins.

**Conclusion:**

Our findings added newer insights into the multifaceted role played by miR-199a-5p/CCR7 in bladder cancer, prompting for the first time this miRNA/chemokine axis that regulates cell metastasis. The results strongly supported miR-199a-5p as a potential therapeutic agent and diagnostic marker of bladder cancer.

## Background

Bladder cancer, a major cause of morbidity and mortality worldwide, is the fourth most common cancer in men. Approximately 25 % of bladder cancer cases are muscle invasive which is more aggressive and with a much worse prognosis [1, 2]. Due to the introduction of cisplatin-based combination chemotherapy, the survival among patients with locally advanced bladder cancer has been improved in the past 30 years. While the recurrence and mortality is still high to those with muscle-invasive bladder cancer. Besides, patients with recurrent or metastatic bladder cancer just remains 14–15 months with cisplatin-based chemotherapy [3–5]. Thus, inhibition of the metastasis is considered to be a promising strategy for bladder cancer therapy.

CCR7, a member of the G-protein-coupled hepta-helical receptors (GPCR) family, is substantially expressed on naive T cells, B cells, and mature dendritic cells [6–8]. Previous studies have demonstrated CCR7 overexpression correlates with lymphatic metastasis and poor prognosis in breast cancer, colon cancer, prostate cancer and gastric cancer [9–12]. In our previous study, we also have found that CCR7 and its ligand CCL21 promote T24 cell proliferation and enhance its migration and invasion via upregulation MMP-2 and MMP-9 [13].

Recently, microRNAs, a class of 19–24 nucleotides small non-coding RNAs, have been proved to be involved in pathogenesis of cancer by either degradation of mRNA or inhibition of its translation [14, 15]. In bladder cancer, miRNAs also have been found to play a crucial role in its development, progression and metastasis, such as miR-27a, miR-143, and miR-150 [16–18]. However, to the best of our knowledge, the relationship between miR-199a-5p and CCR7 in bladder cancer has not been reported to date.

In this study, the miR-199a-5p expression was first identified to be significantly downregulated in human bladder cancer clinical samples and cell lines by qRT-PCR. We found that there was a positive correlation between 199a-5p downexpression and lymphatic metastasis. Overexpression of miR-199a-5p significantly inhibited cell migration and invasion through decreasing the expression of CCR7.

## Methods

### Patients and samples

Forty fresh bladder cancer tissue samples and paired adjacent normal tissue samples were obtained from patients who had undergone transurethral bladder tumor resection or radical cystectomy at Department of Urology, Zhejiang Provincial People’s Hospital after surgical resection. All collected tissue samples were immediately frozen in liquid nitrogen and stored at −80 °C until further use. The institutional ethics committee of Zhejiang Provincial People’s Hospital approved the study, and written informed consent was also obtained from all patients.

### Cell lines and cell culture

The human bladder cancer T24 cell line and human normal bladder epithelial cell line SV-HUC-1 were purchased from the Shanghai Institutes for Biological Sciences, the Chinese Academy of Sciences (Shanghai, China). The T24 and SV-HUC-1 were maintained at 37 °C, 5 % CO_2_ in DMEM (Invitrogen, Carls-bad, CA, USA) or F12K medium (Invitrogen, Carlsbad, CA, USA) supplemented with 10 % fetal bovine serum (FBS), respectively.

### RNA isolation and quantitative real-time PCR

Total RNA was extracted using Trizol (Invitrogen) and treated with RNase-free DNase (Qiagen). Power SYBR qPCR and miRNA qRT-PCR detection kit (Applied Biosystems) were performed in a qRT-PCR detection system (ABI, USA) according to the manufacturer’s protocols.

### miR-199a-5p mimics and transfection

miR-199a-5p mimics and the negative control were obtained from Biomics Biotechnology Inc (Nanjing, China). Transfections were performed using Lipofectamine™ 2000 Reagent (Invitrogen, CA, USA) according to the protocol.

### Wound-healing assay

Cell migration activity was evaluated by wound healing assay. After 48 h transfection, T24 cells were wounded by scraping of three parallel lines with a 200 ml pipette tip, and then washed three times with PBS and incubated in serum-free medium. The wounds were photographed at 0 and 24 h after wounding under a microscope.

### Matrigel invasion assay

Matrigel invasion assay was carried out to assay the cells invasive capacity with a chamber containing a polycarbonate membrane (8 μm pore size) and coated with a layer of extracellular matrix gel (BD Biosciences) according to the manufacturer’s instructions. After 72 h transfection, the number of cells that penetrated the matrigel was counted from six randomly selected fields per membrane.

### Adhesion assay

Microtiter wells were coated with matrix gel (BD Biosciences) at 37 °C, 4 h. Then the wells were blocked for 1 h with 1 % BSA in PBS. After 48 h transfection, 10^5^ cells/well were seeded in serum-free medium. After 1 h adhesion, non-adherent cells were washed away with PBS. The colorimetric MTT-assay was used to determine the number of adherent cells.

### Luciferase reporter assay

The 3′UTR encompassing the target sequence for miR-199a-5p of CCR7 was synthesized and cloned into pMiR-Report vector (Ambion) at HindIII and SpeI sites. After 48 h transfection, luciferase activity was measured using a Glomax 96 luminometer (Promega) according to the manufacturer’s protocols.

### Western blot

After 48 h transfection, cells were harvested and lysed. Proteins were separated by 10 % SDS-PAGE, transferred to PVDF membranes (Millipore, USA) and blotted with primary antibodies (rabbit anti-CCR7, 1:1000, Abcam, USA; rabbit anti-MMP-9, 1:1000, Abcam, USA; rabbit anti-E-cadherin, 1:1000, Abcam, USA; rabbit anti-Vimentin, 1:5000, Abcam, USA; rabbit anti-β-actin, 1:1000, Abcam, USA) at 4 °C overnight followed by incubation with horseradish peroxidase labeled secondary antibodies for 2 h at room temperature.

### Statistics

Data are expressed as means ± SD. Clinicopathological parameters were compared using the χ^2^ test, and continuous variables were compared using the Student’s t-test. *P* value < 0.05 was considered significant.

## Results

### miR-199a-5p expression was downregulated and CCR7 expression was upregulated in human bladder cancer tissue samples and cell lines

**Fig. 1:**
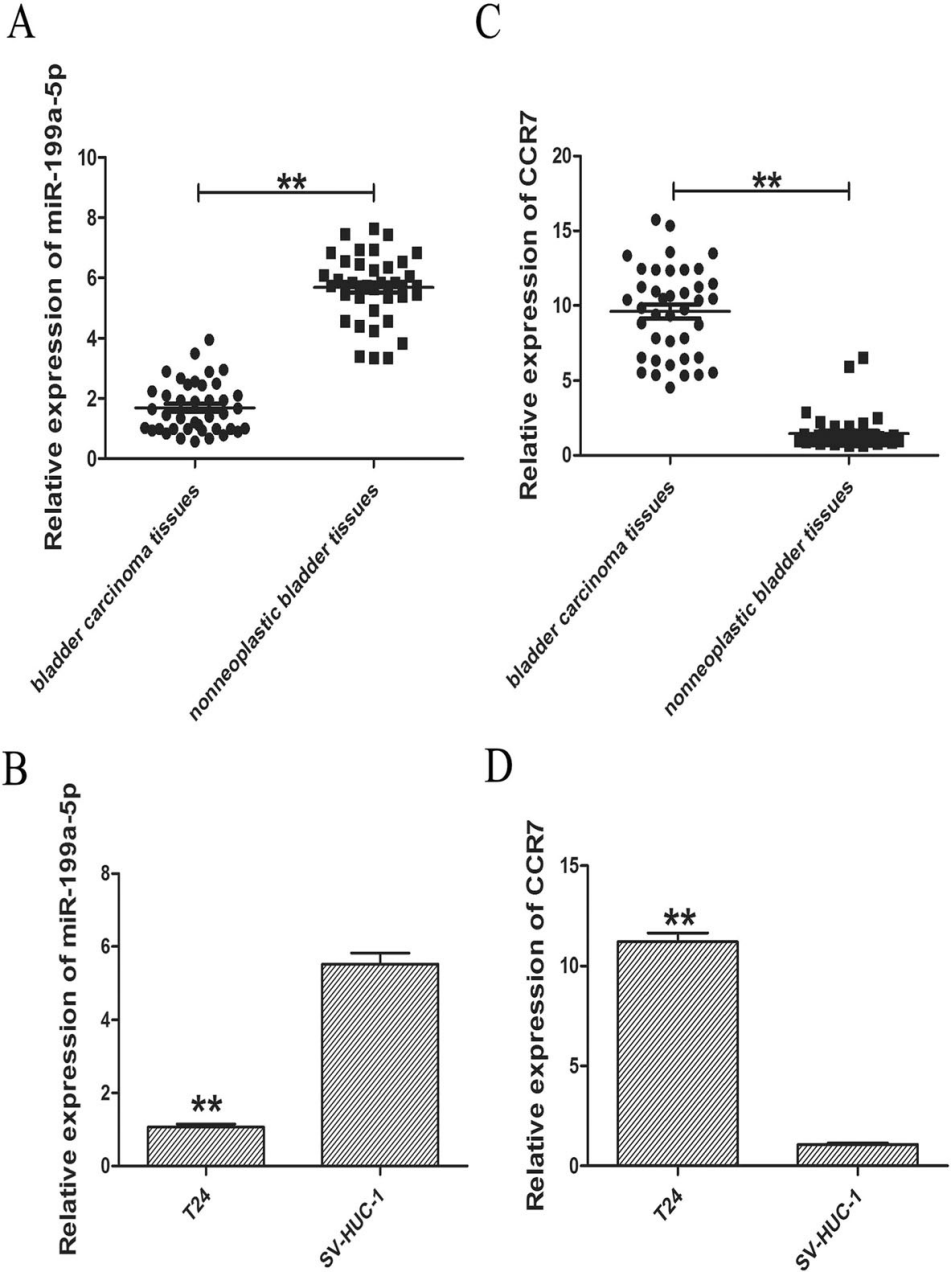
The expression of miR-199a-5p and CCR7 in human bladder cancer tissue samples and cell lines. **a** Relative expression of miR-199a-5p in bladder cancer tissues compared with corresponding normal tissues. **b** Relative expression of miR-199a-5p in bladder cancer T24 cells and human normal bladder epithelial cell (SV-HUC-1). **c** Relative expression of CCR7 in bladder cancer tissues compared with corresponding normal tissues. **d** Relative expression of CCR7 in bladder cancer T24 cells and human normal bladder epithelial cells (SV-HUC-1). Data are expressed as means ± SD of three independent experiments. **P < 0.01 compared with the normal bladder epithelial cell (SV-HUC-1) or normal tissues

To determine whether miR-199a-5p was involved in the tumourigenesis of human bladder cancer, we evaluated the expression levels of miR-199a-5p in human bladder cancer tissue samples and cell lines by qRT-PCR. As shown in Fig. 1a and b, the expression of miR-199a-5p was 3.36-fold and 5.20-fold lower in tumour tissues and bladder cancer cells compared with that in paired adjacent normal tissue or normal cell line SV-HUC-1, respectively. Next, we detected CCR7 expression in human bladder cancer tissue samples and cell lines. Contrary to miR-199a-5p, the expression of CCR7 was 6.60-fold and 10.53-fold higher in tumour tissues and bladder cancer cells compared with that in paired adjacent normal tissue or normal epithelial cell line SV-HUC-1, respectively (Fig. 1c, d).

### Correlation of miR-199a-5p and CCR7 expression with clinicopathological factors

As shown in Table 1, miR-199a-5p downregulation was correlated with TNM stage (*P* < 0.001), Histological grade (*P* = 0.003), Tumor invasion depth (T) (*P* < 0.001) and Lymph node metastasis (N) (*P* < 0.001). Consistent with miR-199a-5p, CCR7 upregulation was also correlated with TNM stage (*P* < 0.011), Histological grade (*P* = 0.001), Tumor invasion depth (T) (*P* < 0.001) and Lymph node metastasis (N) (*P* < 0.001) (Table 2). However, there was no statistical difference between age, gender and tumor size and the miR-199a-5p or CCR7 expression level.

**Table 1:** Correlation between miR-199a-5p expression and clinicopathological characteristics of bladder cancer patients

Characteristics	Total	Expression of miR-199a-5p		*P* value
		High (*n* = 13)	Low (*n* = 27)	
Age				0.758
≤ 60	16	6(37.5 %)	10(62.5 %)	
> 60	24	7(29.2 %)	17(70.8 %)	
Gender				0.316
Male	25	10(40 %)	15(60 %)	
Female	15	3(20 %)	12(70 %)	
TNM stage				<0.001**
0/I	5	3(60 %)	2(40 %)	
II/III/IV	35	10(28.6 %)	25(71.4 %)	
Tumor size				0.501
< 3 cm	16	4(40 %)	12(60 %)	
≥ 3 cm	24	9(37.5 %)	15(62.5 %)	
Histological grade				0.003**
PUNLMP/Low-grade	21	11(52.3 %)	10(47.7 %)	
High-grade	19	2(10.5 %)	17(89.5 %)	
Tumor invasion depth (T)				<0.001**
Tis, Ta, T1	19	12(60 %)	7(40 %)	
T2, T3 or above	21	1(10 %)	20(90 %)	
Lymph node metastasis (N)				<0.001**
N0	12	5(41.7 %)	7(58.3 %)	
N1 or above	28	8(28.6 %)	20(71.4 %)	

*PUNLMP* papillary urothelial neoplasm of low malignant potential; *Low-grade* lowgrade papillary urothelial carcinoma; *High-grade* high-grade papillary urothelial carcinoma (http://www.pathology.jhu.edu/bladdercancer/disease_info.cfm). TNM according to the seventh edition of staging TNM of Union Internationale Contre Le Cancer (UICC) in 2009. ** *P* < 0.01

**Table 2:** Correlation between CCR7 expression and clinicopathological characteristics of bladder cancer patients

Characteristics	Total	Expression of CCR7		*P* value
		High (*n* = 30)	Low (*n* = 10)	
Age				0.368
≤ 60	22	18(81.8 %)	4(18.2 %)	
> 60	18	12(66.7 %)	6(33.3 %)	
Gender				0.234
Male	27	10(37 %)	17(63 %)	
Female	13	3(23.1 %)	10(76.9 %)	
TNM stage				0.011*
0/I	16	8(93.8 %)	1(6.2 %)	
II/III/IV	24	15(62.5 %)	9(37.5 %)	
Tumor size				0.675
< 3 cm	15	8(53.3 %)	7(46.7 %)	
≥ 3 cm	25	22(88.8 %)	3(11.2 %)	
Histological grade				0.001**
PUNLMP/Low-grade	18	10(55.6 %)	8(44.4 %)	
High-grade	22	20(90.9 %)	2(9.1 %)	
Tumor invasion depth (T)				<0.001**
Tis, Ta, T1	17	10(58.8 %)	7(41.2 %)	
T2, T3 or above	23	20(87 %)	3(13 %)	
Lymph node metastasis (N)				<0.001**
N0	12	8(66.7 %)	4(33.3 %)	
N1 or above	28	22(78.6 %)	6(21.4 %)	

*PUNLMP* papillary urothelial neoplasm of low malignant potential; *Low-grade* lowgrade papillary urothelial carcinoma; *High-grade* high-grade papillary urothelial carcinoma (http://www.pathology.jhu.edu/bladdercancer/disease_info.cfm). TNM according to the seventh edition of staging TNM of Union Internationale Contre Le Cancer (UICC) in 2009. ** *P* < 0.01

### Overexpression of miR-199a-5p inhibited the migratory and invasive activity of bladder cancer cells

**Fig. 2:**
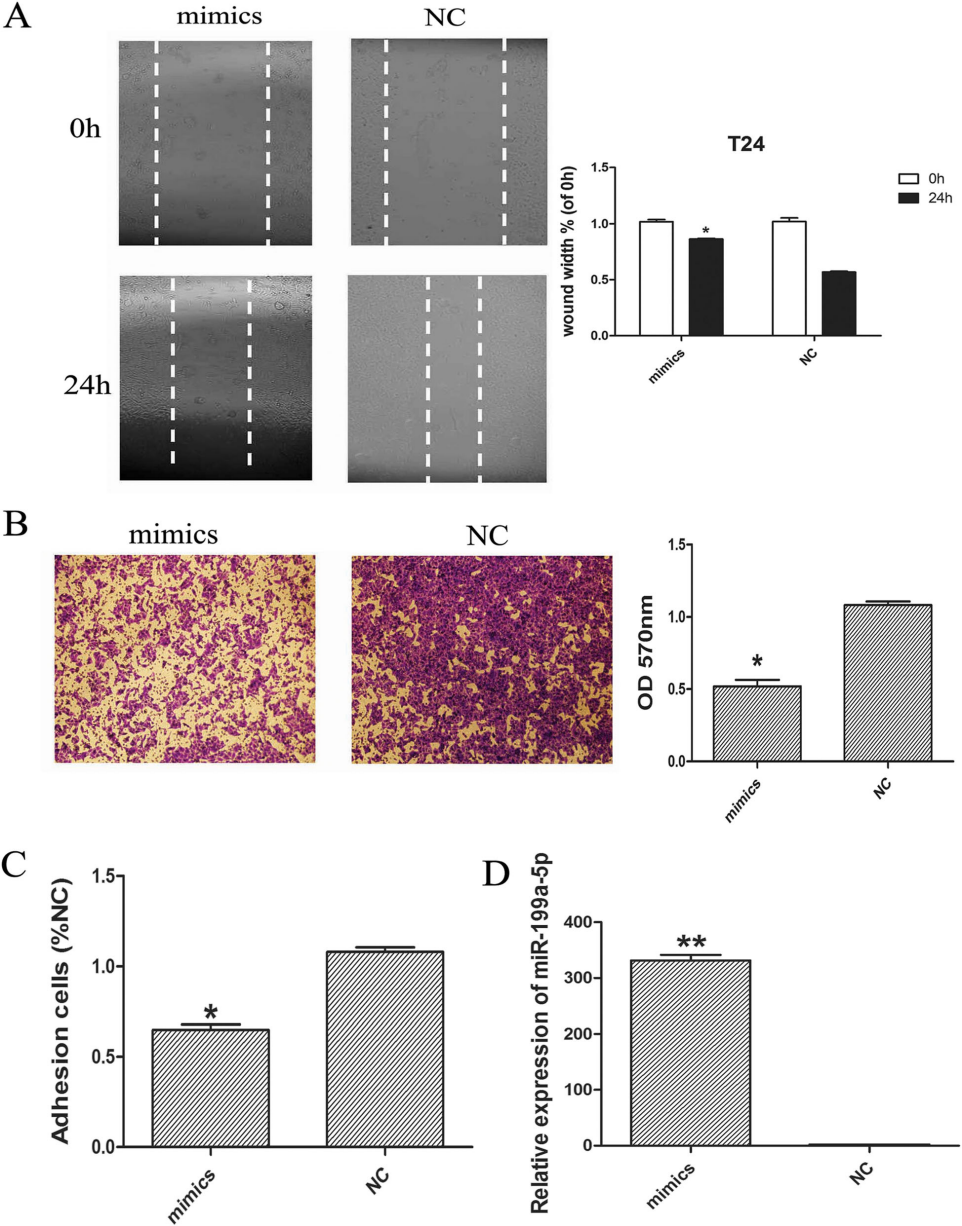
Overexpression of miR-199a-5p inhibited the migratory and invasive activity of bladder cancer cell. **a** Wound-healing assay for bladder cancer T24 cells transfected with miR-199a-5p mimics or the negative control. **b** Representative micrographs (left) and quantification (right) of the Matrigel invasion assay in T24 cells. Magnification × 200. **c** Effects of miR-199a-5p mimics on adhesion to matrix gel of T24 cells. **d** The efficiency of transfection was confirmed by qRT-PCR. Data are expressed as means ± SD of three independent experiments. * *P* < 0.05 compared with the negative control group

In order to examine the effect of miR-199a-5p on the metastasis of baldder cancer cells, we overexpressed miR-199a-5p in T24 cells. The efficiency of transfection was confirmed by qRT-PCR (Fig. 2d). Wound-healing assay showed a significant reduction of cell migration in miR-199a-5p-transfected cells compared with the negative control (Fig. 2a). In addition, Matrigel invasion assay showed that the number of invasive cells in the miR-199a-5p-transfected group was significantly decreased compared with that in the negative control group (Fig. 2b). Moreover, after transfected with miR-199a-5p, the adhesion activity was also decreased in T24 cells (Fig. 2c).

### miR-199a-5p directly targeted the 3′UTR of CCR7 and inhibited the expression of MMP-9 and EMT-related proteins in T24 cells

**Fig. 3:**
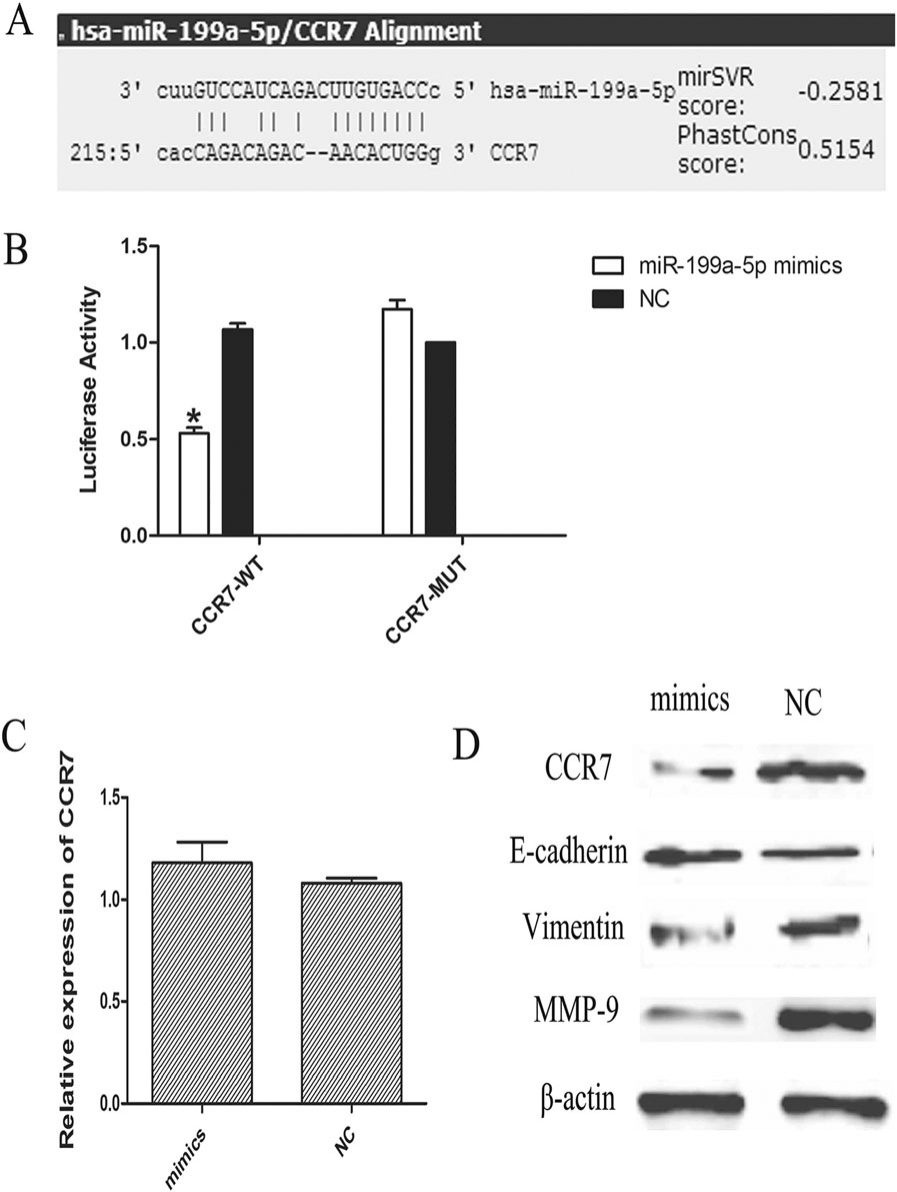
miR-199a-5p directly targeted the 3′UTR of CCR7 and inhibited the expression of MMP-9 and EMT-related proteins in T24 cells. **a** Predicted binding sequences of miR-199a-5p in the 3′UTR of CCR7. **b** Luciferase reporter assays in T24 cells. **c** The gene expression of CCR7 was detected by qRT-PCR in T24 cells. **d** The protein expression of CCR7, E-cadherin, Vimentin and MMP-9 were determined by western blot in T24 cells. Data are expressed as means ± SD of three independent experiments. * *P* < 0.05 compared with the negative control group

To test whether miR-199a-5p could directly interact with CCR7 in bladder cancer cells, we found that the 3′UTR of CCR7 contained a potential miR-199a-5p binding site through bioinformatics software (microRNA.org) (Fig. 3a). As shown in Fig. 3b, after transfected with miR-199a-5p, the relative luciferase activity in the wild-type group was obviously suppressed compared with the mut-type group. Meanwhile, a dramatic decrease of CCR7 was observed in miR-199a-5p-transfected cells compared with the negative control (Fig. 3d). However, after transfected with miR-199a-5p, there was no difference in mRNA level of CCR7 expression (Fig. 3c).

Meanwhile, we also examined the effects of miR-199a-5p/CCR7 on the expression of MMP-9 and EMT-related vimentin and E-cadherin. As shown in Fig. 3d, miR-199a-5p overexpression could significant decrease MMP-9 and vimentin expression, while E-cadherin expression obviously increased in miR-199a-5p-transfected T24 cells compared with the negative control.

## Discussion

In this study, we provided evidence that miR-199a-5p/CCR7 plays an essential role in both the suppression of the EMT process and the metastatic ability of bladder cancer cells. Our results highlighted the importance of the upregulation of the miR-199a-5p in the prevention of the metastatic ability of the bladder cancer cells. On the other hand, we also observed that miR-199a-5p could directly target the 3′UTR of CCR7 and regulate the expression of MMP-9 and EMT-related proteins like vimentin and E-cadherin, eventually suppress the progression of bladder cancer.

In this study, we firstly observed that miR-199a-5p was downregulated in both human bladder cancer tissues samples and bladder cancer cells compared with that in paired adjacent normal tissues or normal epithelial cell line SV-HUC-1 by qRT-PCR, which was consistent with the previous studies [19, 20]. As for the expression of CCR7, there was an inverse relationship with that of miR-199a-5p. In addition, both CCR7 upregulation and miR-199a-5p downregulation were significantly involved in bladder cancer clinicopathological features.

Recent studies have established that miR-199a was significantly abnormally expressed in several solid tumors and functioned as oncogene or tumor suppressor. For instance, miR-199a-3p modulated cell cycle, invasion capability, and doxorubicin sensitivity by targeting mammalian target of rapamycin (mTOR) and c-Met in human hepatocarcinoma cells [21]. Besides, miR-199a-5p was involved in cell proliferation, migration and apoptosis by targeting of multiple myeloma-related angiogenesis [22]. Moreover, miR-199a-5p overexpression promoted cell migration and invasion by targeting klotho in gastric cancer cells [23]. In present study, our results found that overexpression of miR-199a-5p could inhibit bladder cancer cell migration and invasion using wound-healing assay and Matrigel invasion assay.

To explore whether CCR7 was a potential target of miR-199a-5p, we employed luciferase reporter assay and verified this hypothesis. After transfection with miR-199a-5p in T24 cells, the relative luciferase activity was obviously suppressed and CCR7 expression was decreased compared with the negative control. Recent studies suggested that MMP-9 and EMT-related proteins played a crucial role in the process of cell invasion and metastasis that involves loss of cell–cell adhesion and increased cell mobility, and CCR7 might have an effect on metastasis of bladder cancer by regulating MMP-9 and EMT-related proteins [24–28]. Our results showed that overexpression of miR-199a-5p could significant decrease the expression of MMP-9 and vimentin, and obviously enhance the expression of E-cadherin. Taken together, our findings elucidate a novel role of miR-199a-5p in the suppression of cell metastasis through regulation MMP-9 and EMT-related genes by targeting CCR7 in bladder cancer.

Although miR-199a-5p in this study showed a good control to cell metastasis by targeting CCR7 in human bladder cancer, it should be noted that this study only revealed the effect of miR-199a-5p on cell metastasis. Future studies will make great efforts to explore more functional role of miR-199a-5p in the tumorigenesis and progression of human bladder cancer.

## Conclusion

In summary, our present study revealed that downregulation of miR-199a-5p was positively correlated with clinicopathological factors in bladder cancer. Collectively, these results add newer insights into the multifaceted role played by miR-199a-5p/CCR7 in bladder cancer, prompting for the first time this miRNA/chemokine axis that regulates cell metastasis. These data supported miR-199a-5p as a potential therapeutic agent and diagnostic marker of bladder cancer.

## Abbreviations

CCR7: C-C chemokine receptor type 7
EMT: Epithelial-Mesenchymal Transition
GPCR: G-protein-coupled hepta-helical receptors
MMP-9: Matrix metalloproteinases 9
mTOR: Mammalian target of rapamycin
qRT-PCR: Quantitative Real Time PCR
UTR: Untranslated region

## References

[1] Siegel R, Ma J, Zou Z (2014). Cancer statistics, 2014. CA Cancer J Clin.

[2] Kaplan AL, Litwin MS, Chamie K (2014). The future of bladder cancer care in the USA. Nat Rev Urol.

[3] von der Maase H, Sengelov L, Roberts JT (2005). Long-term survival results of a randomized trial comparing gemcitabine plus cisplatin, with methotrexate, vinblastine, doxorubicin, plus cisplatin in patients with bladder cancer. J Clin Oncol.

[4] Grossman HB, Natale RB, Tangen CM (2003). Neoadjuvant chemotherapy plus cystectomy compared with cystectomy alone for locally advanced bladder cancer. N Engl J Med.

[5] Calabrò F, Sternberg CN (2009). Neoadjuvant and adjuvant chemotherapy in muscle-invasive bladder cancer. Eur Urol.

[6] Sallusto F, Lenig D, Förster R (1999). Two subsets of memory T lymphocytes with distinct homing potentials and effector functions. Nature.

[7] Ben-Baruch A (2008). Organ selectivity in metastasis: regulation by chemokines and their receptors. Clin Exp Metastasis.

[8] Ben-Baruch A (2006). The multifaceted roles of chemokines in malignancy. Cancer Metastasis Rev.

[9] Tutunea-Fatan E, Majumder M, Xin X (2015). The role of CCL21/CCR7 chemokine axis in breast cancer-induced lymphangiogenesis. Mol Cancer.

[10] Li J, Sun R, Tao K (2011). The CCL21/CCR7 pathway plays a key role in human colon cancer metastasis through regulation of matrix metalloproteinase-9. Dig Liver Dis.

[11] Heresi GA, Wang J, Taichman R (2005). Expression of the chemokine receptor CCR7 in prostate cancer presenting with generalized lymphadenopathy: report of a case, review of the literature, and analysis of chemokine receptor expression. Urol Oncol.

[12] Arigami T, Natsugoe S, Uenosono Y (2009). CCR7 and CXCR4 expression predicts lymph node status including micrometastasis in gastric cancer. Int J Oncol.

[13] Mo M, Zhou M, Wang L (2015). CCL21/CCR7 enhances the proliferation, migration, and invasion of human bladder cancer T24 cells. PLoS One.

[14] Brennecke J, Hipfner DR, Stark A (2003). bantam encodes a developmentally regulated microRNA that controls cell proliferation and regulates the proapoptotic gene hid in Drosophila. Cell.

[15] Bartel DP (2009). MicroRNA Target Recognition and Regulatory Functions. Cell.

[16] Drayton RM, Dudziec E, Peter S (2014). Reduced expression of miRNA-27a modulates cisplatin resistance in bladder cancer by targeting the cystine/glutamate exchanger SLC7A11. Clin Cancer Res.

[17] Lin T, Dong W, Huang J (2009). MicroRNA-143 as a tumor suppressor for bladder cancer. J Urol.

[18] Ye L, Xi HH, Bin L (2014). miR-150 Modulates Cisplatin Chemosensitivity and Invasiveness of Muscle-Invasive Bladder Cancer Cells via Targeting PDCD4 In Vitro. Med Sci Monit.

[19] Ichimi T, Enokida H, Okuno Y (2009). Identification of novel microRNA targets based on microRNA signatures in bladder cancer. Int J Cancer.

[20] Song T, Zhang X, Yang G (2015). Decrement of miR-199a-5p contributes to the tumorigenesis of bladder urothelial carcinoma by regulating MLK3/NF-κB pathway. Am J Transl Res.

[21] Fornari F, Milazzo M, Chieco P (2010). MiR-199a-3p regulates mTOR and c-Met to influence the doxorubicin sensitivity of human hepatocarcinoma cells. Cancer Res.

[22] Raimondi L, Amodio N, Di Martino MT (2014). Targeting of multiple myeloma-related angiogenesis by miR-199a-5p mimics: in vitro and in vivo anti-tumor activity. Oncotarget.

[23] He XJ, Ma YY, Yu S (2014). Up-regulated miR-199a-5p in gastric cancer functions as an oncogene and targets klotho. BMC Cancer.

[24] Moirangthem A, Bondhopadhyay B, Mukherjee M (2016). Simultaneous knockdown of uPA and MMP9 can reduce breast cancer progression by increasing cell-cell adhesion and modulating EMT genes. Sci Rep.

[25] Mehner C, Hockla A, Miller E (2014). Tumor cell-produced matrix metalloproteinase 9 (MMP-9) drives malignant progression and metastasis of basal-like triple negative breast cancer. Oncotarget.

[26] Zheng H, Takahashi H, Murai Y (2006). Expressions of MMP-2, MMP-9 and VEGF are closely linked to growth, invasion, metastasis and angiogenesis of gastric carcinoma. Anticancer Res.

[27] Voulgari A, Pintzas A (2009). Epithelial-mesenchymal transition in cancer metastasis: mechanisms, markers and strategies to overcome drug resistance in the clinic. Biochim Biophys Acta.

[28] Byles V, Zhu L, Lovaas JD (2012). SIRT1 induces EMT by cooperating with EMT transcription factors and enhances prostate cancer cell migration and metastasis. Oncogene.

